# Relative residential property value as a socio-economic status indicator for health research

**DOI:** 10.1186/1476-072X-12-22

**Published:** 2013-04-15

**Authors:** Neil T Coffee, Tony Lockwood, Graeme Hugo, Catherine Paquet, Natasha J Howard, Mark Daniel

**Affiliations:** 1 Social Epidemiology and Evaluation Research Group, School of Population Health & Sansom Institute for Health Research, Division of Health Sciences, GPO Box 2471, City East Campus, University of South Australia, Adelaide 5001, Australia; 2 Discipline of Geography, Environment and Population and the Australian Population and Migration Research Centre, The University of Adelaide, North Terrace Campus, Adelaide 5000, Australia; 3 Centre for Regulation and Market Analysis, UniSA School of Business, University of South Australia, City West Campus, GPO Box 2471, Adelaide 5001, Australia; 4 Research Centre of the Douglas Mental Health University Institute, 6875 LaSalle Boulevard Montreal, Quebec H4H 1R3, Canada; 5 Department of Medicine, The University of Melbourne, St Vincent's Hospital, Clinical Sciences Building 29 Regent Street, Fitzroy, Melbourne, VIC 3065, Australia

**Keywords:** Socio-economic status, Cardiometabolic risk, Geographic information system, Residential property value, Relative location factor

## Abstract

**Background:**

Residential property is reported as the most valuable asset people will own and therefore provides the potential to be used as a socio-economic status (SES) measure. Location is generally recognised as the most important determinant of residential property value.

Extending the well-established relationship between poor health and socio-economic disadvantage and the role of residential property in the overall wealth of individuals, this study tested the predictive value of the Relative Location Factor (RLF), a SES measure designed to reflect the relationship between location and residential property value, and six cardiometabolic disease risk factors, central obesity, hypertriglyceridemia, reduced high density lipoprotein (HDL), hypertension, impaired fasting glucose, and high low density lipoprotein (LDL). These risk factors were also summed and expressed as a cumulative cardiometabolic risk (CMR) score.

**Methods:**

RLF was calculated using a global hedonic regression model from residential property sales transaction data based upon several residential property characteristics, but deliberately blind to location, to predict the selling price of the property. The predicted selling price was divided by the actual selling price and the results interpolated across the study area and classified as tertiles. The measures used to calculate CMR were collected via clinic visits from a population-based cohort study. Models with individual risk factors and the cumulative cardiometabolic risk (CMR) score as dependent variables were respectively tested using log binomial and Poisson generalised linear models.

**Results:**

A statistically significant relationship was found between RLF, the cumulative CMR score and all but one of the risk factors. In all cases, participants in the most advantaged and intermediate group had a lower risk for cardio-metabolic diseases. For the CMR score the RR for the most advantaged was 19% lower (RR = 0.81; CI 0.76-0.86; p <0.0001) and the middle group was 9% lower (RR = 0.91; CI 0.86-0.95; p <0.0001) than the least advantaged group.

**Conclusions:**

This paper advances the understanding of the nexus between place, health and SES by providing an objective spatially informed SES measure for testing health outcomes and reported a robust association between RLF and several health measures.

## Background

### Residential property as an SES measure

Residential property may well be the most valuable asset owned by many individuals and can provide the basis for a residential property wealth indicator reflecting socio-economic status (SES). Di and colleagues (2003) [1] reported that home equity accounted for 21% of household net wealth in the United States of America (USA). For low SES households the percentage of household wealth represented by residential property was substantial, accounting for approximately 50% of household net wealth. Property represents a significant proportion of an economy’s gross domestic product (GDP) [2]. Rothenberg and colleagues (1991) [3] stressed that a society’s wellbeing is dependent on a fundamental understanding of housing market structures.

The three most important contributors to residential property value are, “location, location, location” [4]. Location matters and by association, so does the associated social geography. Social geography not only describes composition [5] but, and of importance to this research, includes the associated spatial variation.

One of the key features of residential property as a traded commodity is its immovability or location specific capital, and this provides the basis of making location a prime residential property value determinant [6]. While residential property supply and demand is often expressed using economic equilibrium, the addition of location extends this concept to include spatial equilibrium where proximity and location influences price [7].

How best to model residential property value requires an understanding of how more or less desirable residential property locations can be used as a meaningful indicator of local area SES. The locational aspect of residential property and the acknowledgement that a group of residential properties may be described as more or less valuable is often described as a residential property market. While the notion of a residential property market composed of a number of interrelated submarkets is a cornerstone of real estate transactions, the literature is still undecided on the best methodology to determine the spatial boundaries of such submarkets [8]. The themes expressed in the literature converge in the recognition that submarkets are best defined using spatial and structural identifiers [8]. It is also acknowledged that submarkets should be derived from data rather than on the basis of some *a priori* definition such as suburbs or postcodes [9]. Such data should reflect the underlying residential property real estate market structure of the area under study and not rely on residential property characteristics such as size, style, age, number of bedrooms [4,10,11].

Residential property market structure cannot be identified by property characteristics and socio-economic geography alone. The identification of a residential property market structure also requires the expression of price (market) to give it an economic entity status [12]. Identifying all of the attributes contributing to the underlying market structure is a challenge, as the list of locational attributes is extensive [13]. Locational attributes often serve as a ‘proxy’ for the numerous unobserved attributes affecting residential property value [14]. A methodology described by Gallimore et al., (1996) [15] isolating the effects of location to the error term of an hedonic regression model simplifies the need to account individually for such numerous attributes while still capturing their effect on value. This is achieved by describing price in terms of observable residential property characteristics only, remaining deliberately ‘blind’ to locational characteristics and interpreting the error term as a proxy for location. This provides a methodology for determining the relative value of location to the study area mean by interpreting the relationship between residential property value and SES as the nexus between ‘where you live’ (place) rather than the ‘absolute value of the residential property you live in’ and SES as being an important relationship when studying health outcomes. This is what the Relative Location Factor (RLF) [16] was designed to reflect. Of importance for this study is that the resulting interpolated continuous RLF surface can be assigned to any residential property. This allows analyses to be potentially free of the modifiable area unit problem (MAUP) [17] and provide a better understanding of any local spatial variation that may be occurring within the traditionally presented spatial units. MAUP is an issue associated with scale and configuration of spatial units such that statistical associations may change as the size of the spatial unit changes (scale) or as the study area is subdivided into different spatial configurations (zonation). Even though this issue has been described by geographers for a number of years [18-21], few health studies acknowledge, let alone account for, MAUP despite the burgeoning use of place in health research.

### Health and socioeconomic status

SES has long been established as one, if not the most, important population health risk factor [22] with pioneering work in the 19th Century by Louis-Rene Villerme [23], Rudolf Virchow [24], Charles Booth [25]. Charles Booth’s 1898–99 maps of poverty in London highlighted the link between poor health and poverty and incorporated a spatial dimension. Since this early work there are few health outcomes that have not been associated with SES. Studies have investigated SES and mortality [26-30], respiratory diseases [31,32], chronic diseases [27,33-37], obesity levels [38-42], oral health [43] and health-related behaviours such as smoking [39,44-47], and alcohol consumption [48,49]. This association is often reported as a gradient, with social position strongly influencing health outcomes such that across many disease or behaviours the effects are more prevalent as SES decreases [50]. These numerous studies provide a significant literature that has repeatedly associated low SES with poorer health.

SES is a complex, multidimensional concept that is typically represented using one or all of the “triad” of indicators, education, income and occupation. Beyond the many studies that use these three SES measures, other researchers have represented SES in terms of housing tenure [47,51,52], housing type [42,53], number of bedrooms [32,52,54], overcrowding measures [37,55], number of offspring [55-57], car ownership [51,52,55,57], and asset or wealth measures [33,43,58-60].

In addition, efforts have been directed towards developing integrated SES indices, such as the United Kingdom Index of Deprivation [61,62] or the Australian Socio-Economic Index for Areas (SEIFA) [63-66]. Such measures are however, constructed for predetermined spatial geographies at the time of the census. Of particular note when using Census derived SES indices, is that many of the spatial unit boundaries change from one census to the next, methodologies and input variables change, and there is limited temporal comparability. In the case of the Australian SEIFA index, the Australian Bureau of Statistics specifically warns against comparisons of SEIFA from one census to the next [63].

SES measures are collected either via survey or derived from area level data from population census collections. Both forms of data capture have strengths and weaknesses. Survey data are subject to limitations associated with recall, incorrect responses, non-responses and socially desirable responses [67-69], especially with questions on income and education. Area level data, collected via a national census can provide population level data, but are similarly subject to the above limitations in addition to being susceptible to the MAUP [17].

The potential limitations associated with SES measures makes them unsuitable for understanding local variations within spatial units. Despite the many studies researching health and SES, the lack of an understanding about the spatial distribution of SES is still a major challenge. Many studies rely on SES data presented as an average for a predetermined aggregated spatial unit. MAUP [17] may be introduced into the analysis as is the potential to mask any SES variations within these spatial units. This raises the question as to which spatial unit is the most appropriate when analysis results may be different depending on the choice made.

One area of research investigating SES measures that are less prone to MAUP is the use of residential property or housing value. In health research this is an emerging literature linking residential property value and SES, including the use of Council Tax Valuation Bands (CTVB) in the United Kingdom (UK) [70] and property values in the USA [41,71]. The UK study [70] used the eight band CTVB and reported associations between many health and lifestyle outcomes and SES expressed as property value classes. A study in the USA used residential property value to test the relationship between obesity and area level SES in Seattle [71]. Results from the Seattle study indicated that the residential property level measure was more predictive than area-level SES in identifying fair or poor health status [71]. Another USA study reported an association between property value and obesity, such that women were 3.4 times more likely to be obese if they lived in the bottom quartile than the top quartile [41]. Australian studies have shown how the variation in socio-economic indicators was correlated with the variation in median house price movement when aggregated to the same spatial unit [72,73]. RLF [16] adds to this emerging area of research and provides a relative location wealth SES measure for social and health researchers.

Based on the long standing relationship between poor health and SES and the significance of residential property in the overall wealth of an individual, this study tested the predictive value of RLF as a measure of SES to six clinical measures of chronic disease risk and a cumulative risk score.

## Methodology

### Study area

**Figure 1:**
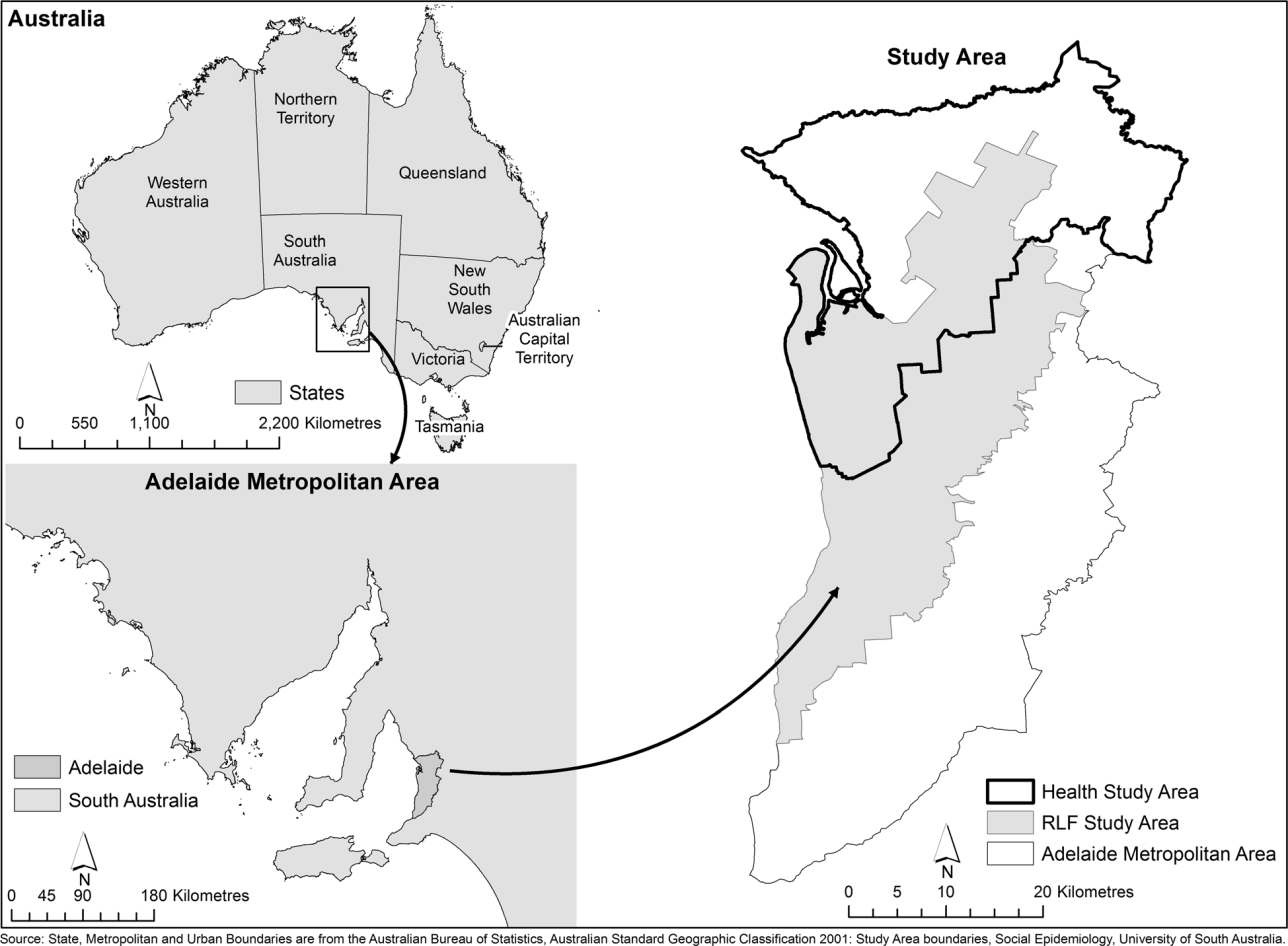
Study Area.

The study area for calculating RLF was the Adelaide Metropolitan Area which stretches approximately 80 kilometres north–south and 30 kilometres east–west (Figure 1) and had a population in 2001 of 1.07 million [74]. The study area for the health data was the North Western and Northern Adelaide Metropolitan Area, stretching approximately 60 kilometres north–south and 30 kilometres east–west with a 2001 population of 410341, 38% of Adelaide’s metropolitan population [74].

### Participants

This study utilised data from the North West Adelaide Health Study (NWAHS) which was established to provide a longitudinal population-based biomedical cohort for investigating a number of chronic conditions and health-related risk factors over three waves of data collected between 2000 and 2010. This report involves a cross-sectional analysis that used the first wave (W1) of NWAHS data collected between 2000 to 2003 with 4056 adults who were 18 years of age or older. All participants were randomly selected via from the Adelaide White Pages Telephone Directory [75]. Data collection included self-report socio-demographic data, clinical and biomedical data, and prescription medication usage via linkage with the Australian Pharmaceutical Benefits Scheme. Participants provided their residential address during the self-report data collection and this information was validated during the clinic visit. Of 4056 participants recruited for W1, 4041 supplied valid addresses. All NWAHS participants provided written consent to use their health and residential address data. Geographic information system (GIS) software was used to geocode the participants’ address for spatial analysis. Ethics approval was granted by the Human Research Ethics Committees of the University of South Australia, the North West Adelaide Health Service, and the South Australian Department of Health.

### Relative location factor

The method to derive the RLF was described in detail in an earlier paper [16] and had three main steps. Step one specified a global hedonic regression model using residential property sales transaction data based upon several residential property characteristics, but deliberately blind to location, to predict the selling price of the residential property (Table 1).

**Table 1:** RLF model input data

**Variable**	**Type**	**Description**
Sale Price (SP)	continuous	Sale Price in dollars obtained from the South Australian Government
Dwelling size (DS)	continuous	Equivalent main area in square metres (source: South Australian Valuer General).
Dwelling age (DA)	continuous	Age in years obtained (source: South Australian Valuer General)
Dwelling land area (LA)	continuous	Area in square metres taken from the digital cadastre (source: South Australian Valuer General)
Dwelling style(DT)	Dummy	If “South Australian Housing Trust style” or “poor conventional” = 1 else 0 (source: South Australian Valuer General)
Dwelling quality(DQ)	Dummy	If high quality based on housing style i.e. “high quality contempory”; or “high quality conventional” or “high quality ranch” or “mansion” or “architectural design“ = 1 else 0 (source: South Australian Valuer General)

Source: Coffee and Lockwood, 2012.

**Figure 2:**
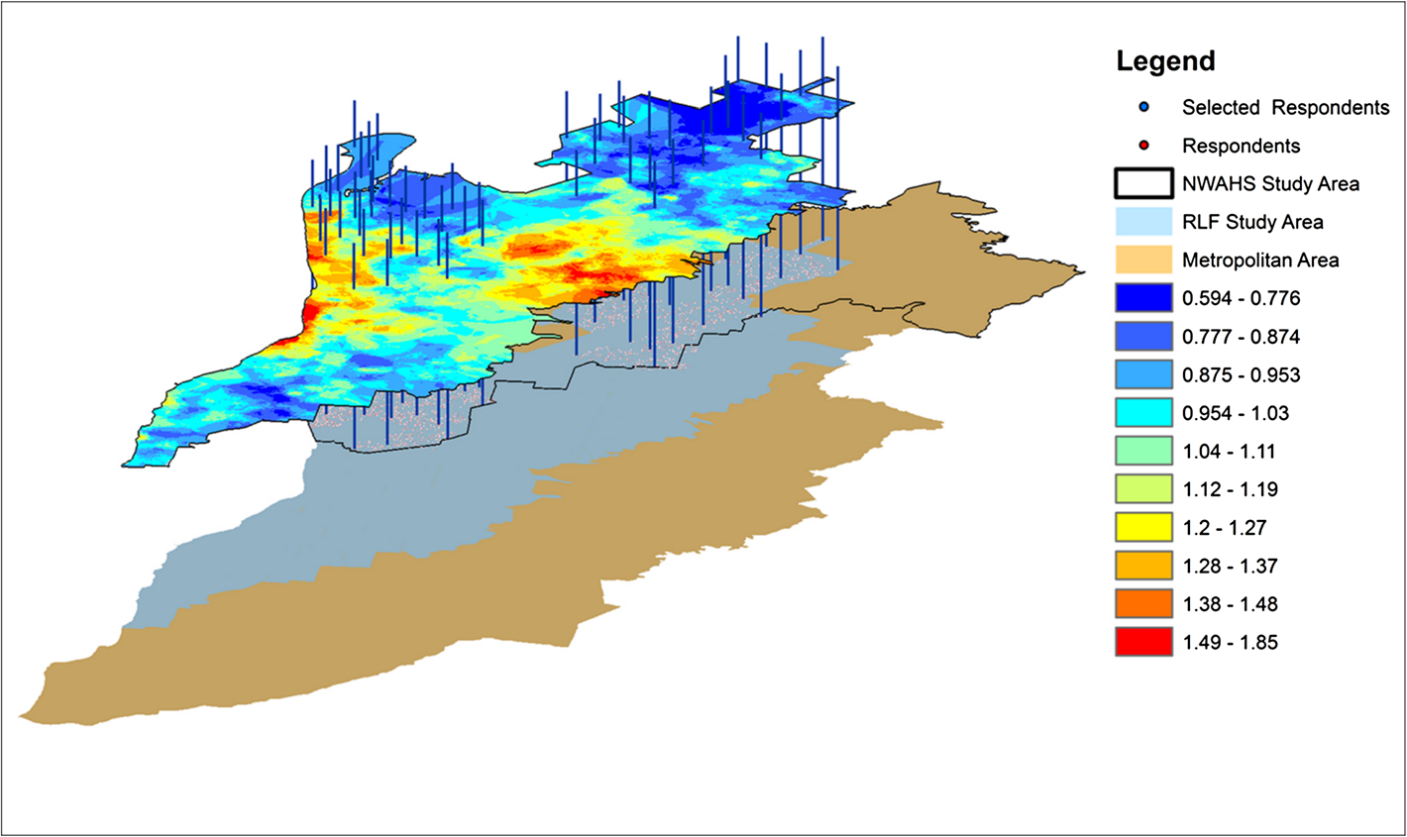
Linking RLF to respondent location.

This enabled the model error to be inferred as a proxy for the omitted variable bias of any attributes describing the influence of market value due to location. Such error was expressed as the ratio of the predicted price to actual price. Only Sales Transaction data in the study area that had been assessed as representing market value by the South Australian Valuer General were used in this stage (n = 6800). Sale transactions between May and October 2001 were used to ensure market comparability. In step 2, the RLF was created using GIS to interpolate a continuous raster surface representing the individual residential property predicted to actual price ratios (a value of one accorded to the mean ratio, a value less than one was interpreted as location lowering the residential property values and a value greater than one was interpreted as location positively influencing residential property values). Step 3 used GIS to extract the value from the RLF surface to the geocoded respondent’s location (Figure 2). The RLF resolution was set at 25 metres to more closely approximate the individual residential property level. RLF was only calculated for urban areas as different factors influence urban and non-urban (semi-rural or rural) residential property markets (Figure 1). RLF was grouped in tertiles using the ESRI Fisher-Jenks natural breaks algorithm [76]. This method for classifying RLF was used to provide groupings that were more meaningful and represented groups where the between group variation was maximised and the within group variation was minimised.

### Measures

#### Cardiometabolic risk factors and score

In this study, the health measures analysed were six clinical risk factors and a cumulative cardiometabolic risk score. This was calculated as the sum of the six clinical risk factors. The risk factors were selected to reflect components of the metabolic syndrome and were based on internationally established clinical cut-offs for expressing metabolic syndrome and cardiometabolic risk generally. The risk markers were defined by the International Diabetes Federation (IDF) [77] and included:

• hypertension (systolic blood pressure ≥130 mmHg or diastolic blood pressure ≥ 85 mmHg or treated for hypertension with medication);

• abdominal adiposity (waist circumference ≥ 94 cm in men and ≥ 80 cm in women);

• reduced high density lipoprotein (HDL) cholesterol (< 1.03 mmol/L in men and < 1.29 mmol/L in women);

• raised triglycerides level (≥ 1.7 mmol/L or treated for lipid abnormality with medication); and

• raised fasting plasma glucose (≥ 5.6 mmol/L or previously diagnosed diabetes).

A sixth marker, increased low density lipoprotein (LDL) cholesterol levels (≥ 4.1 mmol/L), was included based on a cut-off from the National Cholesterol Education Program (NCEP) ATP-III [78]. Each risk marker was scored as either zero (below the cut-off) or one (above the cut-off) except for reduced HDL which was zero (above the cut-off) and 1 (below the cut-off). CMR was calculated by summing the six risk markers and the value ranged from 0 (no risk markers) to 6 (all risk markers). Each clinical risk factor was assumed to contribute equally to total cardiometabolic risk, therefore no weighting was applied.

#### Covariates

Covariates included participant reported age, gender and education. The NWAHS participant’s age and gender were collected during the phone recruitment process and level of education was collected from the self-report questionnaire. Age was modelled in 10 year increments and education was dichotomised as with or without a university education.

#### Statistical analysis

Models for each risk factor as the dependant variable were tested using log binomial generalized linear models. The CMR score as the dependent variable was tested using Poisson regression. Parameter estimates were exponentiated to provide relative risk (RR). All analyses included participants’ gender, age and education. The analyses were conducted in SAS (version 9.2; SAS Institute Inc, Cary, North Carolina). Statistical significance was set at alpha = 0.05.

## Results

### Sample characteristics

Of the 4041 participants with a geocoded address, 3915 had a complete cardio-metabolic risk profile. This number was reduced by a further 330 participants who lived in semi-urban fringe or rural locations as RLF was not calculated in these locations. The final sample after removing the non-urban NWAHS participants was 3585. Characteristics of the sample are presented in Table 2.

**Table 2:** Descriptive characteristics of the Individual Survey Sample (n = 3585)

**Characteristic**		**N (%)**
Gender: Male		1731 (48.2)
Female		1862 (51.8)
Age (Mean(SD))		50.4 (16.3)
Education: No university degree		3155 (87.8)
University graduate		438 (12.2)
Relative Location Factor (Mean (SD))	Tertile 1	0.76 (0.084)
	Tertile 2	0.95 (0.046)
	Tertile 3	1.21 (0.146)
Central Obesity	Yes	2332 (65.0)
	No	
		1253 (35.0)
Hypertriglyceridemia (or medication)	Yes	1221 (34.1)
	No	2364 (65.9)
Reduced HDL	Yes	1054 (29.4)
	No	2531 (70.6)
Hypertension	Yes	1897 (52.9)
	No	1688 (47.1)
Diabetes Risk or diagnosed diabetic	Yes	798 (22.3)
	No	2787 (77.7)
High LDL	Yes	629 (17.8)
	No	
		2898 (82.8)
Cardiometabolic risk score (Mean (SD))		2.2 (1.5)

Table 3 displays the results from the analysis testing for associations between RLF, the six risk factors and the cardiometabolic risk (CMR) score. Five of the six risk factors were significantly associated with RLF. Participants in the most advantaged tertile had a lower risk of having central obesity, hypertriglyceridemia, reduced HDL, hypertension, and impaired fasting glucose compared with the most disadvantaged tertile. Higher LDL was not statistically significantly associated with RLF. RLF was a strong predictor of the likelihood of poorer cardiometabolic health in the lowest SES grouping. The CMR score was also statistically significantly associated with RLF. Participants in the most advantaged and middle RLF tertile respectively had a 19% and 9% lower CMR score than participants in the most disadvantaged RLF tertile. All covariates were statistically significant in most of the models, with the exceptions of the LDL model where only age was statistically significant and the HDL model where only gender was statistically significant.

**Table 3:** Parameter estimates for associations between RLF and cardiometabolic risk factors and cardiometabolic risk score (n = 3585)

**Tertiles (Natural breaks)**		**RR**	**95% CI**		**P**
Central Obesity***	RLF: 3 v 1	0.89	0.83	0.95	0.0004
	RLF: 2 v 1	0.93	0.89	0.98	0.0033
Hypertriglyceridemia***	RLF: 3 v 1	0.79	0.70	0.90	0.0005
	RLF: 2 v 1	0.90	0.82	0.98	0.0173
Reduced HDL#	RLF: 3 v 1	0.79	0.67	0.92	0.0025
	RLF: 2 v 1	0.87	0.78	0.97	0.0159
Hypertension***	RLF: 3 v 1	0.94	0.88	1.01	0.0824
	RLF: 2 v 1	0.90	0.85	0.95	<.0001
Diabetic\diabetes Risk***	RLF: 3 v 1	0.52	0.43	0.64	<.0001
	RLF: 2 v 1	0.79	0.70	0.89	<.0001
High LDL^	RLF: 3 v 1	0.95	0.77	1.17	0.6277
	RLF: 2 v 1	1.05	0.90	1.23	0.5399
CMR Score***	RLF: 3 v 1	0.81	0.76	0.86	<.0001
	RLF: 2 v 1	0.91	0.86	0.95	<.0001

Gender, Age and Bachelor Education were included in all models.

*** Gender, Age and Bachelor Education Significant.

**#** Gender Significant.

**^** Age Significant.

## Discussion

Six cardiometabolic risks and a cumulative CMR score were modelled to test the predictive power of RLF. In all but one of the risk factors and for the cumulative cardiometabolic risk, RLF was statistically significantly associated with the likelihood of poorer health in the most disadvantaged group relative to the middle and most advantaged groups. The CMR score of a participant living in the most advantaged tertile was 19% lower than a participant in the most disadvantaged tertile and 9% lower for the middle group compared to the most disadvantaged group.

The RLF methodology outlined in this paper and provided in more detail in an earlier paper [16] provides an alternative or complementary, objective SES measure for place and health research. While SES is a many dimensioned and complex concept, RLF is a measure of relative residential property value, which as reported by Di and colleagues [1] represented between 21-50% of an individual’s wealth. The relative nature of RLF provided the link between ‘where you live’ and residential property wealth as the important relationship when developing an individual residential property SES measure. It is imperative when deriving a relative measure such as the RLF to have it relate to a larger area such as Metropolitan Adelaide, so that people’s choice of places to live includes as many competitive properties as possible. This makes the “relative” component of the measure more realistic in terms of the significance of location.

The utility of the RLF construction methodology is reflected through its ability to be generated at any time, subject to the availability of analysed residential property price transaction data together with the corresponding residential property characteristics data at the time of sale. This makes the methodology suitable for any local, state or national jurisdiction that collects sales transactions data. In addition to Australia, many European countries collect sales transactions as a core component of their Land Administration System [79]. Others, such as the USA collect these data as an integral component of their property taxation system [41]. As long as the residential property address is included the data can be geocoded enabling linkage with other relevant data within a GIS environment. This makes RLF an SES measure which can be constructed for many local, national and international jurisdictions and allow for meaningful comparisons of SES and health associations. Few of the traditional SES measures can be compared internationally due to different classifications and\or collection methodologies. In addition to the applicability to any country that collects spatially enabled residential property sales data, this methodology can be applied to specific point-of-time or longitudinal studies as well as to varied spatial extents adding to its versatility and applicability.

A specific aim of this study was to add to the discussion within the growing literature that recognises residential property wealth as a SES measure. This paper has expressed residential property wealth as a function of relative location value rather than the absolute value of the property itself and found a significant link with cardiometabolic risk. This overcomes the problem of two neighbours having significantly different absolute property values while both belonging, in the SES sense, to similar, if not the same, SES sub group, removing the potential for distortion due to specific residential property differences. Conversely, two residential properties in different locations may have the same absolute value but attract different RLF scores providing a more reliable indication of SES through an inherently better underlying sense of ‘place’. RLF was an objective SES measure and as a relative location factor for residential property value overcomes the potential challenges of MAUP and enabled the local SES variation to be captured. This study adds to the small but growing number of studies investigating the use of residential property value for SES and the application of GIS methods to link disparate data using location [41,70,71].

This is the first study in our knowledge to use residential property sales data to interpolate a continuous relative value surface and apply this as a SES measure to evaluate associations with cardiometabolic health risks. As noted above, the majority of analyses linking SES with health rely on predetermined aggregate spatial units.

RLF provides an objective SES measure that emphasises ‘relative location value’ rather than the ‘residential property value’ lived in. This approach can contribute to the overall advancement in the use of GIS regarding place and health research by expressing the importance of residential property wealth as a complementary SES metric.

## Conclusion

RLF was statistically significantly associated with a lower CMR score and a lower risk of being centrally obese, having hypertriglyceridemia, reduced HDL, hypertension or being at risk of or diagnosed with diabetes. These results add to the long standing association between SES and poorer health conditions, supported a gradient of poorer health with declining SES, and provided an objectively-derived residential property wealth based measure that could be applied with any study using individual participant address data. While many studies have concentrated on the health association with SES, few studies have looked beyond education, income and occupation. These are important indicators, but an objective measure that reflects both residential property wealth and location provides the basis for overcoming MAUP.

One of the enduring issues with many place and health studies is the lack of rigour associated with the choice or appropriateness of spatial boundaries. Such studies tend to focus on the rigor in selecting health data, accounting for bias and ensuring appropriate statistical methodologies. While these are all vitally important aspects of any study, the expression of place requires a similar level of attention and should be subject to similar levels of scrutiny.

RLF is a very flexible measure and can be interpolated for any jurisdiction that has location based residential property sales data with associated residential property characteristics. These sales transaction data are recorded in most jurisdictions as part of the land administration systems. Unlike statistical agency measures, RLF can be calculated quarterly, half-yearly, annually or for any period supported by residential property sales transaction data. In addition, it is not limited to census collection years and can be used to measure SES change over time as well as over space. This paper advances the understanding of the nexus between place, health and SES by providing an objective spatially informed measure for testing health outcomes and reported a robust association between RLF and cardiometabolic risk.

## Abbreviations

CMR: Cardiometabolic risk; CTVB: Council Tax Valuation Bands; GDP: Gross domestic product; GIS: Geographic information system; HDL: High density lipoprotein; LDL: Low density lipoprotein; MAUP: Modifiable area unit problem; NWAHS: North West Adelaide Health Study; RLF: Relative Location Factor; RR: Relative risk; SEIFA: Socio-Economic Index for Areas; SES: Socio-economic status; UK: United Kingdom; USA: United States of America; W1: Wave one data collection.

## Competing interests

The authors declare that they have no competing interests.

## Authors’ contributions

NC and TL Conception of the project; data capture, spatial and statistical analysis and interpretation; writing the manuscript; and important critical review of the intellectual content. CP and NH: Data capture, health data definition, statistical analysis and critical review of the intellectual content. GH and MD: important critical review of the intellectual content and final approval of the version to be published. All authors read and approved the final manuscript.
